# XGB Modeling Reveals Improvement of Compressive Strength of Cement-Based Composites with Addition of HPMC and Chitosan

**DOI:** 10.3390/ma17020374

**Published:** 2024-01-11

**Authors:** Duygu Ege, Ali Reza Kamali

**Affiliations:** 1 Institute of Biomedical Engineering, Boğaziçi University, Rasathane Cd, Kandilli Campus, Kandilli Mah., Istanbul 34684, Turkey; 2 Energy and Environmental Materials Research Centre (E2MC), School of Metallurgy, Northeastern University, Shenyang 110819, China; a.r.kamali@cantab.net

**Keywords:** methyl cellulose, carboxymethyl cellulose, cement, compressive strength, graphene oxide, zoledronic acid, extreme boost modeling, chitosan

## Abstract

This study investigates the improvement in the compressive strength of cellulose/cement-based composites. Methyl cellulose (MC), carboxymethyl cellulose (CMC), and hydroxypropyl cellulose (HPMC) are separately used as the cellulose phase with different wt%. Graphene oxide (GO) and zoledronic acid (ZOL) are used as additives for bone regeneration for various formulations. Utilizing Extreme Gradient Boosting (XGB) modeling, this research demonstrates the roles of the choice of the cellulose phase, wt% of cement phase, % gelatin, % citric acid, degradation time, and concentration of GO and ZOL in influencing compressive strength. The XGB regression model, with an R^2^ value of 0.99 (~1), shows the predictive power of the model. Feature importance analysis demonstrates the significance of cellulose choice and the addition of chitosan in enhancing compressive strength. The correlation heatmap reveals positive associations, emphasizing the positive influence of HPMC and CMC compared with MC and the substantial impact of chitosan and citric acid on compressive strength. The model’s predictive accuracy is validated through predicted compressive strength values with experimental observations, providing insights for optimizing cellulose-reinforced cements and enabling tailored material design for enhanced mechanical performance.

## 1. Introduction

In recent decades, there has been a notable focus on bioresorbable cements, for the restoration and substitution of compromised bone tissues. Bioresorbable cements have gained significant attention owing to their ability to occupy bone voids, eliminating the necessity for metallic implants [1]. A key advantage lies in the natural degradation of these implants at the implantation site, eliminating the need for subsequent surgeries to extract the implants post-healing, which distinguishes them favorably from non-degradable alternatives [2,3].

Calcium phosphate cements (CPC) are the focus of extensive research due to their outstanding physical, mechanical, and biological properties [4,5,6]. Because of their biocompatibility, bioactivity, biodegradability, and osteoconductivity, there is significant interest in advancing their development. CPCs can be injected and, subsequently, solidify in vivo, conforming to the shape of the defect site. Among the most commonly utilized bioceramics for CPC production are dicalcium phosphate dihydrate (DCPD, CaHPO_4_.2H_2_O), calcium sulfate dihydrate (CSD; CaSO_4_·2H_2_O), and tetra-calcium phosphate (TTCP, Ca_4_(PO_4_)_2_O) [5,6,7]. Upon injection of a mixture of TTCP and DCPD into the defect site, it undergoes transformation into hydroxyapatite (HA) [5,6,8].

To modify the rheological characteristics, such as injectability, setting temperature, and mechanical properties, CPC may be incorporated into a polymeric matrix. The inclusion of a polymeric matrix can also facilitate the penetration of body fluids into bone substitutes, thereby supporting three-dimensional cell migration, cell growth, and ultimately ossification [5,9]. In this context, the selection of an appropriate polymer is crucial for optimizing these functions [10,11,12,13,14,15]. Cellulose stands out as a promising polymer for this purpose; however, its lack of water solubility, attributed to intra-molecular hydrogen bonding, limits its applicability in biomedical contexts [16,17,18]. Consequently, hydrophilic and water-soluble derivatives of cellulose have been developed [19]. Methylcellulose (MC), carboxymethyl cellulose (CMC), and hydroxypropyl cellulose (HPMC) are cellulose ether derivatives and have undergone extensive research for biomedical applications [10,11]. Gelatin is also added to the CPC composites due to its arginine, glycine, and aspartic amino acid sequence (RGD) groups, which improve cell–biomaterial interactions and also enhance mechanical properties. Nanocomposites, particularly those involving functionalized cellulose/gelatin-reinforced cements, have attracted significant interest in the biomaterials and tissue engineering field due to their potential for enhanced mechanical properties and biocompatibility [12,13]. Citric acid is also incorporated into the CPC/polymeric composites to improve their mechanical performance [1]. Citric acid (CA) stands out as a promising candidate because of its carboxylic groups, which establish a network with CMC chains and amide bonds with gelatin. Furthermore, citrate ions are also present in the bone mineral; therefore, they are non-toxic and, additionally, relatively inexpensive [7,14].

Nanocomposites incorporating graphene have garnered significant attention in the field of biomedical applications owing to their exceptional physical, electrical, and chemical attributes, as well as their extensive surface area and high chemical purity. Consequently, integrating graphene and its derivatives with calcium phosphate cements (CPC) represents a promising approach to enhance various aspects of CPC performance, including mechanical properties, stability, workability, and osteoconductivity, as well as cell adhesion, proliferation, and differentiation [15,16].

Research indicates that introducing bisphosphonates (BPs) to injectable bone substitutes (IBS) can modify the physicochemical characteristics of the IBS, including hardening time, morphology, porosity, and mechanical properties, all within acceptable ranges [17]. Specifically, the addition of BPs has been observed to slightly reduce the mechanical properties of the IBS samples with added BPs [18,19,20]. Boanini et al. [21] conducted a comparison of the in vitro effects of zoledronic acid (ZOL) and alendronate using hydroxyapatite (HA) nanocrystals. ZOL exhibited a higher affinity for the HA structure compared to alendronate, although both showed a similar level of osteoclast apoptosis. The strong affinity of ZOL for HA was attributed to their favorable structural compatibility. Additionally, in vivo studies on BP-loaded calcium phosphate cement (CPC)-based scaffolds have substantiated the enhancement of bone formation through BP incorporation [20,21].

The foundation of this investigation lies in studying the data of methyl cellulose (MC)/cement, carboxymethyl cellulose (CMC)/cement, and hydroxypropyl cellulose (HPMC)/cement-based composites, wherein the focus is given to the concentrations of MC, CMC, HPMC, GO, citric acid, cement, ZOL, cellulose, and chitosan, as well as degradation time. The significance of each component’s contribution to the resulting mechanical properties is assessed through a comprehensive computational modeling approach. Using an advanced machine learning algorithm, specifically the Extreme Gradient Boosting (XGB) regression model, enables a deeper understanding of the complex relationships within these nanocomposites, offering predictive insights into their compressive strength [22,23]. The integration of computational modeling in this study is crucial not only for predictive purposes but also for unraveling the underlying mechanisms governing the mechanical behavior of cellulose-reinforced cements. Through an array of analyses, including correlation heatmaps, feature importance assessments, and Shapley Additive Explanation (SHAP) techniques, this study aims to elucidate the hierarchical impact of each parameter on compressive strength [24]. Such insights are invaluable for the optimization of biomaterials, laying the groundwork for the development of mechanically robust and functionally tailored nanocomposites for tissue engineering applications.

## 2. Materials and Methods

### 2.1. Data Collection

In this study, data are collected from six previous papers, and 68 datasets are collected [7,25]. Following this, the mechanical properties of the composites were covered for different formulations. The eight independent variables in this study include choice of cellulose phase as either MC, CMC, or HPMC; % of cellulose; wt% of cement; wt% of citric acid; wt% of gelatin; wt% of GO; ZOL concentration; and degradation time (in days). The dependent variable is compressive strength. Table 1 below shows the composition of the biomaterials. Detailed parameters are provided in Table 1 in Supplementary Table S1. 

### 2.2. Computational Modeling

The implementation of machine learning algorithms in this study was conducted using Python 3.9, leveraging key libraries such as Pandas, Numpy, Scipy, Matplotlib, Seaborn, and Scikit-learn. Specifically, the analysis involved the application of an XGB regression model [27]. The Python codebase for this study is accessible at https://github.com/duyguege/machine-learning.git (accessed on 15 October 2023).

#### 2.2.1. XGB Regressor

The XGB regressor, developed by Guestrin and Chen in 2016, stands as an ensemble gradient-boosting algorithm [28,29]. This model enhances predictive capabilities by sequentially incorporating trees, creating a robust learner from weaker ones [29]. Predictions are then derived by aggregating scores from individual leaf nodes [30]. Renowned for its efficiency and accuracy, XGB is a prevalent choice in regression tasks. To mitigate over-fitting to outliers, the model applies a second-order Taylor expansion to the loss function and normalizes the objective function [28,29,30,31].

#### 2.2.2. Training, Hyper-Tuning, and Validation Processes

The dataset underwent division into training (80%) and test (20%) sets, with the former used for model development and the latter for evaluation. The Scikit-learn library in Python facilitated the application of models. Optimal parameters for the XGB model were determined through hyperparameter tuning, considering factors such as the subsample ratio of columns, number of estimators, maximum depth, and learning rate (shrinkage factor). To ensure a balance between underfitting and overfitting, optimal parameters were selected for both training and test sets [32]. Performance evaluation involved 10-fold cross-validation, with the model ultimately used for predicting stiffness values [33,34].

#### 2.2.3. Correlation Heatmap

A correlation heatmap, generated using the Seaborn module in Python, served to evaluate the relationships among independent factors (choice of CMC, HPMC, or MC; degradation time; % of cement; wt% of GO; % citric acid; % chitosan; and ZOL concentration) and the dependent variable, compressive strength. A higher correlation coefficient (r) indicated multicollinearity among the independent variables [22]. MC is coded as 0, CMC is coded as 1, and HPMC is coded as 2 in the dataset to analyze their correlation. A value above 0.8 indicates a very strong correlation and a value between 0.6 and 0.8 indicates a strong correlation. A value between 0.4 and 0.6 shows that there is a moderate correlation. A value between 0.2 and 0.4 demonstrates a weak correlation. If the value is below 0.2, there is only a very weak correlation. Finally, a negative value indicates a negative correlation, and the relevant strength of values is also true for negative correlation values.

#### 2.2.4. Feature Importance

Feature importance was assessed by calculating the significance of features through an integrated function in the Scikit-learn implementation of the XGB model. Features were ranked based on their importance [23,35].

#### 2.2.5. Model Performance Assessment

The success of the models was gauged based on higher coefficients of determination (R-squared or R^2^) with lower root mean square error (RMSE) and mean absolute error (MAE) indicative of superior performance. Model assessment encompassed the evaluation of R^2^, RMSE, and MAE [22,23,24].

#### 2.2.6. Shapley Additive Explanation

The Shapley Additive Explanation (SHAP) technique, introduced by Lundberg and Lee in 2017, was employed for unraveling complex relationships in machine learning models [36]. Utilizing SHAP as a Python model interpretation tool, this study delved into the marginal relationship between predicted compressive strength values and each feature. SHAP values elucidated the contribution of each feature to compressive strength prediction, with negative and positive values signifying negative and positive contributions, respectively. The SHAP summary plot depicted the impact of each parameter on compressive strength, with the primary y-axis displaying SHAP values and the secondary y-axis featuring a color bar indicating high feature values.

## 3. Results and Discussion

In this study, we use XGB boosting to study the effect of processing parameters on the compressive strength of cellulose/cement-based composites. The mechanical properties of these biocomposites are influenced by many factors and the choice of the components is critical. This study explores how material choice intricately shapes the mechanical characteristics of cellulose/cement composites. The investigated factors are ZOL concentration; wt% of GO, wt% of cement, wt% of citric acid, wt% of chitosan, and choice of cellulose.

**Figure 1:**
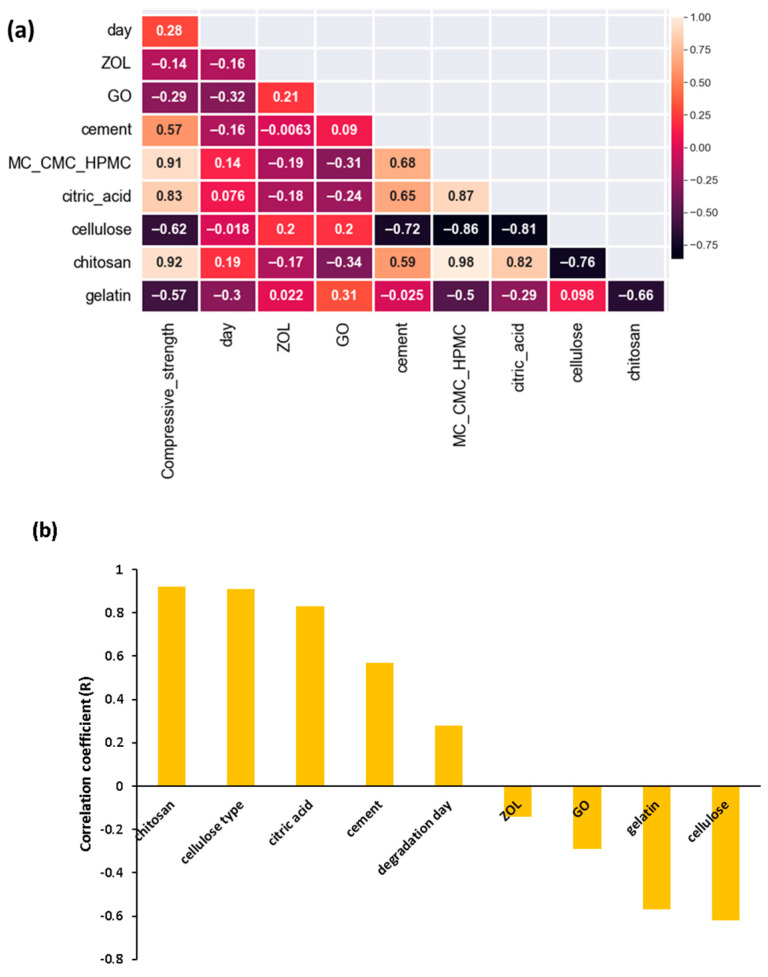
Relationship between parameters. (**a**) Correlation heatmap for compressive strength, independent parameters degradation time (day), ZOL and GO concentration, % of cement phase (cement), and whether the choice of polymer is MC, CMC, or HPMC. (**b**) Correlation coefficient (R) of the components and dependent variable, compressive strength.

Figure 1a shows the correlation heatmap for the dependent variable (compressive strength), ZOL concentration, wt% of GO, wt% of cement, wt% of citric acid, wt% of chitosan, and choice of cellulose (MC_CMC_HPMC) as the cellulose phase; Figure 1b shows the correlation coefficient ® of the parameters and the dependent variable.

**Figure 2:**
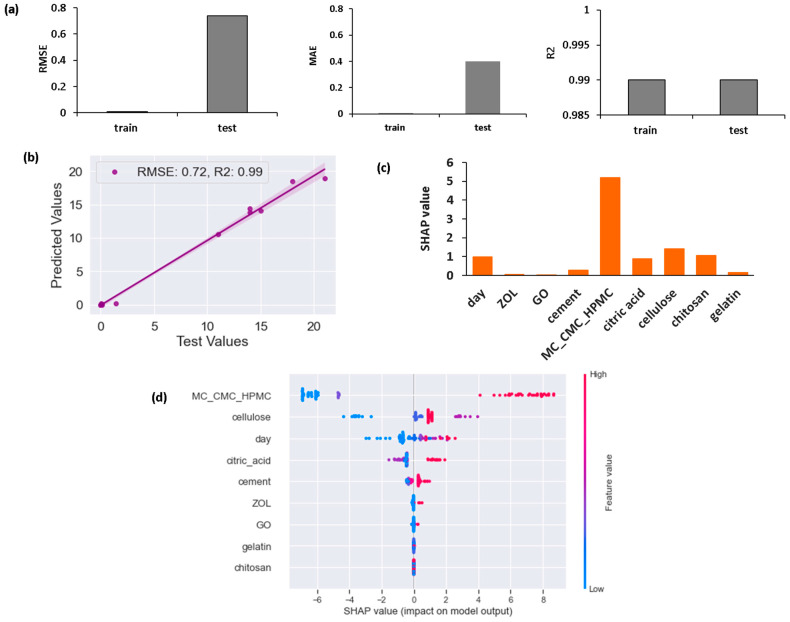
XGB model for compressive strength and (**a**) RMSE, R^2^, and MAE of training and testing for compressive strength; (**b**) predicted and observed compressive strength; (**c**) SHAP values for each feature; (**d**) SHAP values for independent variables on prediction of compressive strength.

In the model, MC, CMC, and HPMC are coded as 0.1 and 2, respectively. The correlation coefficient ranged from −1 to 1. Meghanathan et al. [37] indicate that a value from 0.8 to 1 for the correlation coefficient reveals a very strong correlation and a value from 0.6 to 0.8 indicates a strong correlation. A value between 0.4 and 0.6 shows that there is a moderate correlation. A value between 0.2 and 0.4 demonstrates a weak correlation. If the value is below 0.2, there is only a very weak correlation. As there is a strong positive correlation (correlation coefficient = 0.79) for the choice of cellulose (MC_CMC_HPMC) parameters with compressive strength, this indicates that CMC leads to higher compressive strength than MC, and HPMC leads to higher compressive strength than CMC. Citric acid and chitosan concentration also have a strong positive correlation with compressive strength. Zhang et al. [38] incorporated chitosan in β-tricalcium phosphate and observed significant improvement in mechanical properties. This was explained to be due to the complexation of the calcium ions of the calcium phosphate phase with the functional groups of chitosan. Citric acid improves mechanical properties by interacting with hydroxyl groups of the carboxymethyl or hydroxypropyl methyl cellulose phases, which forms an ester crosslink [10]. Citric acid also has a “salting-out” effect, which increases the packing of the cements and reduces porosity [39]. The % of cement has a comparatively weaker positive correlation with compressive strength. For degradation time (days), there is a positive correlation of 0.28. This means that, generally, degradation increases compressive strength; however, as this value is low, this means that there is only a weak correlation. The correlation coefficients for ZOL and GO are quite low and negative, and this shows that they have a weak correlation with the compressive strength of the composites. This means that their effect on compressive strength is much less effective. The literature indicates that GO increases compressive strength significantly when added in a polymeric phase due to hydrogen bonding; however, this effect is found to be much less pronounced in cements [25,40,41]. An increase in the concentration of cellulose and gelatin is also observed to have a negative correlation. Figure 2 shows (a) RMSE, R^2^, and MAE for training and testing compressive strength data, (b) predicted and observed (test) compressive strength values, (c) SHAP values for each feature, and (d) SHAP values for independent variables for the prediction of compressive strength.

**Figure 3:**
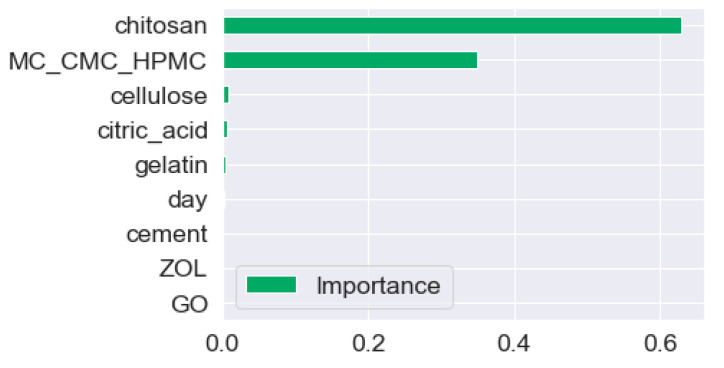
Feature importance implemented from XGB model.

Figure 2 shows that R^2^ for the XGB model is 0.99 (~1). This is an excellent fit of the test values to the model trained by the training dataset [42]. R^2^ for testing and training are also very similar, which shows that the model works similarly for training and testing data. However, RMSE and MAE values are higher for testing data than for training data. This shows that there is a degree of overfitting of the model for the dataset. Despite this, the RMSE and MAE values are quite low even for test data (MAE = 0.4, RMSE = 0.7); therefore, the model can still provide satisfactory knowledge for analyzing the importance of each factor and the mechanical behavior of the composites [42]. Figure 2c shows that SHAP values are positive for all the features. This shows that the model gives predictions with higher values than experimental data. The highest SHAP value is observed for the choice of cellulose. According to Figure 2d, higher values of the dataset for % cement phase, wt% citric acid, wt% of cellulose, and degradation time increase the SHAP value. This shows that higher values of % of cement, wt% citric acid, wt% of cellulose, and degradation time improve the compressive strength prediction. For other data values (GO, ZOL, chitosan, gelatin), the effect is not as distinct. Figure 3 shows the feature importance implemented from the XGB model.

**Figure 4:**
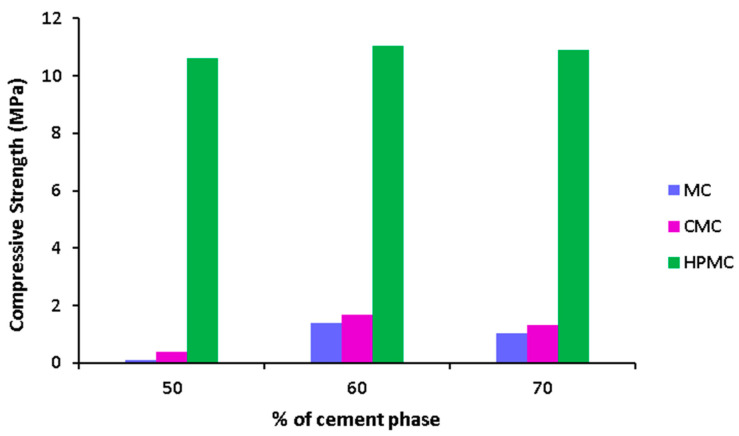
Predicted compressive strength versus % of cement phase for (4% cellulose and 0% chitosan).

According to Figure 3, the most important factor affecting compressive strength is the addition of chitosan. Chitosan’s positive amine groups may interact via ionic bonds with negatively charged carboxyl groups of cellulose. It may also form complexes with the cement phase, improving the mechanical strength [43]. The choice of type of cellulose is also found to be effective. HPMC leads to higher compressive strength than CMC, and CMC is observed to lead to higher compressive strength than MC. This could be due to the presence of the hydroxypropyl group, which improves the solubility of the cellulose phase, which leads to a more homogeneous blending of cellulose with the cement and gelatin phase [7,14,44]. The wt% of cellulose, citric acid, and cement are observed to have much less importance on compressive strength. As citric acid can form amide bonds with gelatin and also crosslinks with HPMC, CMC, and MC, it increases the compressive strength of the composites; however, this effect is quite insignificant in comparison to chitosan and cellulose components. GO, ZOL, gelatin concentrations, and degradation time are observed to have a much less significant effect on compressive strength than the other factors. Figure 4 shows predicted compressive strength for various % of the cement phase.

**Figure 5:**
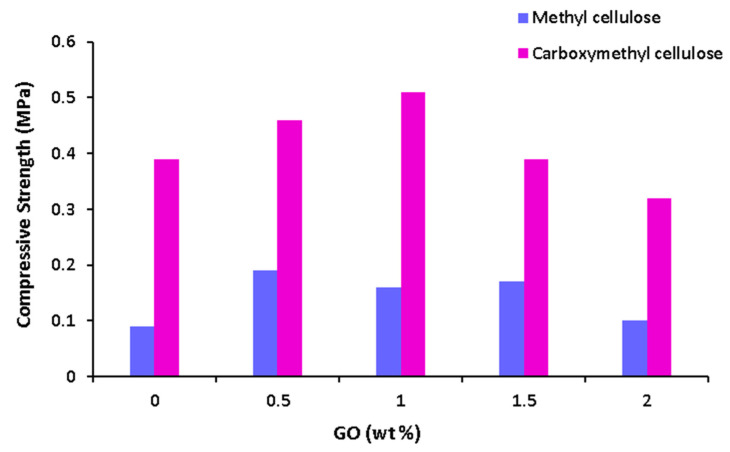
Predicted compressive strength versus wt% of GO (cement phase = 50%, % cellulose = 4, % chitosan = 0).

According to Figure 4, with the increase of compressive strength, compressive strength increases. More distinctly, HPMC leads to higher compressive strength following CMC and MC. The higher compressive strength is because of the presence of ionic interactions between negatively charged carboxyl and hydroxyl groups of HPMC and positive amine groups of the gelatin phase. Moreover, the cement phase and CMC may also interact via ionic interactions [7,25,45]. On the other hand, MC has hydrophobic interactions and hydrogen bonding occurs between MC chains [1]. This shows that the presence of ionic interactions leads to a composite with higher strength than MC. Figure 5 shows the predicted compressive strength for various wt% of GO.

**Figure 6:**
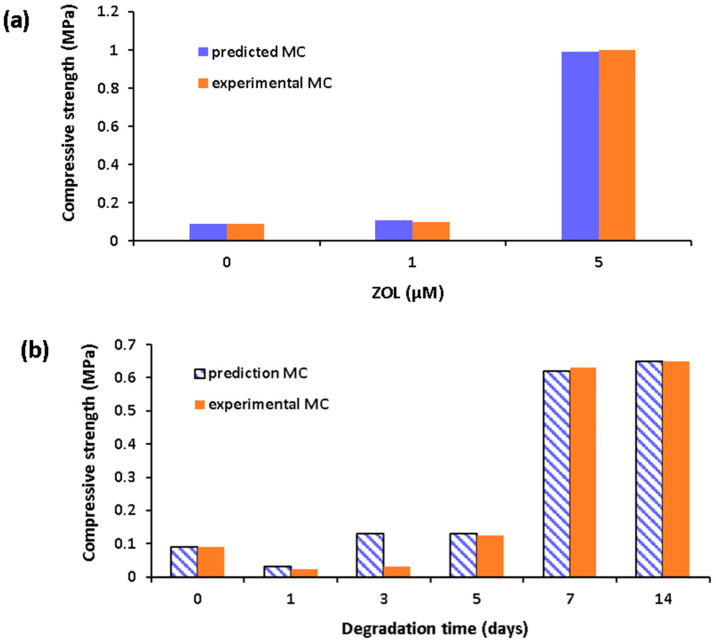
Predicted and experimental compressive strength values versus (**a**) concentration of ZOL and (**b**) degradation time (days) (cement phase = 50%, % cellulose = 4, % chitosan = 0).

GO’s hydroxyl, carboxyl, and epoxy groups carboxylate ions of CMC to form hydrogen bonding. This interaction increases the strength of the biomaterial [45]. According to Figure 5, GO increases compressive strength up to 1 wt% loading in CMC. This dosage goes down to 0.5 wt% for MC. This is possibly because of the easier mixing of GO in CMC than MC due to CMC’s carboxyl groups. Above these concentrations, compressive strength decreases, and this is because of the agglomeration of GO at higher loadings. This trend is frequently observed in research papers [25,46,47]. Moreover, the increase here is not very significant compared to the effect of the choice of cellulose type. This is also previously shown in Figure 3, as GO was shown to have little effect on compressive strength. In Figure S1, the predicted values and experimental values are provided for GO and MC-added 50 wt% cement biocomposites. According to this figure, the values are quite similar for most of the predictions and experimental groups for studied compositions. The difference was relatively more pronounced for the addition of 0.5 and 1.5 wt% of GO. The difference was 5 and 6% off for 0.5 and 1.5 wt% of GO-added samples; however, even this difference was considerably small. Figure 6 shows predicted and experimental values of the compressive strength for various concentrations of ZOL and degradation time.

According to Figure 6a, ZOL minimally increases compressive strength with a concentration of 1 µM; however, with an increase of concentration to 5 µM, an abrupt increase in compressive strength was observed. As seen in Figure 6b, degradation initially reduces compressive strength; however, with further degradation time, compressive strength increases. This is because the cement phase hardens over time, significantly increasing the compressive strength on day 7 [1]. When experimental values and predictions are compared, it is observed that values are quite similar except for day 3. A higher prediction is observed compared to the experimental value. This increase may also be explained by the positive SHAP values, which indicate that predictions are higher than experimental values.

Overall, according to this study, many parameters are demonstrated to be effective on compressive strength. The most effective parameters are the addition of chitosan and the choice of cellulose phase. The RMSE and MAE values for the test set are greater than those for the training set, indicating the presence of overfitting. However, the difference is not substantial. Therefore, the obtained results appear to be meaningful. Nonetheless, conducting additional machine learning studies in the future with larger datasets would be valuable to further analyze relationships between parameters.

For example, for the compressive modulus and % strain at break, there is not enough data for the prepared composites. Once there is enough data in the literature, the effect of the studied parameter may also be applied to analyze their effect on compressive modulus and % strain at break. Additionally, as can be observed in Table 1, the initial concentration of gelatin and cellulose is not the same. This may affect the outcome of the model. In the future, it would be more effective if initial concentrations are kept the same for gelatin, citric acid, and cellulose to be able to compare the effect of the type of cellulose on mechanical strength. Despite this, the data obtained from the XGB modeling is fruitful and open doors for future studies to further optimize the mechanical properties of the cellulose/cement-based composites. Since the dataset on stiffness and % strain were more constrained so far, we could only study compressive strength. In the future, it would also be beneficial to study the effect of each parameter on the stiffness and % strain of the prepared composites.

## 4. Conclusions

The XGB regression model exhibited strong predictive capabilities with an R^2^ value of 0.99, underscoring the pivotal roles of many factors influencing the mechanical properties of the cements. Feature importance analysis emphasized the significant contributions of cellulose choice and chitosan to the observed improvements in compressive strength. The predictive accuracy of the model was validated through the congruence of predicted compressive strength values with experimental observations, providing actionable insights for the optimization of cellulose-reinforced cements. These findings offer a valuable foundation for tailored material design, allowing for the enhancement of mechanical performance in cellulose-reinforced biocomposites.

## Figures and Tables

**Table 1:** Wt% of each component in the prepared composites.

Choice of Cellulose	wt% of Gelatin in Polymeric Phase (wt%)	wt% of Citric acid in Polymeric Phase (wt%)	wt% of Cellulose in Polymeric Phase (wt%)	Bioceramic Phase in (Liquid Phase + Bioceramic Phase) (%)	Bioceramic Phase CSD/(TTCP+ DCPD)	(Ref.)
**MC (in polymer)**	2.5	3	8	0, 20, 30, 50	25/75	[1,20,26]
**CMC (in polymer)**	10	20	2	62.5, 65, 67.5, 70	20/80	[7,25]
**HPMC**	0	20–40	0–4	64.3	20/80	[10]

## Data Availability

The original contributions presented in the study are included in the article and supplementary material, further inquiries can be directed to the corresponding author.

## Supplementary Materials

The following supporting information can be downloaded at: https://www.mdpi.com/article/10.3390/ma17020374/s1, Figure S1: Compressive strength of predicted and experimental MC loaded samples versus wt% of GO; Table S1: Compressive strength of the samples in relation to the composition of the ceramic-based composites.
