# Supplementary material for: XGB Modeling Reveals Improvement of Compressive Strength of Cement-Based Composites with Addition of HPMC and Chitosan
Source: Materials (Basel). 2024 Jan 11;17(2):374. doi: 10.3390/ma17020374 (PMC10820120; doi:10.3390/ma17020374)
Supplement: Supplementary file 1 [file materials-17-00374-s001.zip › materials-2746746-supplementary.pdf]

Supplementary

Table S1 shows the compressive strength of the samples in relation to the composition of the ceramic-based composites.

Table S1. Compressive strength of the samples in relation to the composition of the ceramic-based composites

Compressive strength (MPa)	Degradation (day)	ZOL (μM)	% GO	% cement	Cellulose phase	% citric acid	% cellulose	% chitosan	Ref.
0.006	1	0	0	0	0	3	8	0	[1]
0.006	3	0	0	0	0	3	8	0	[1]
0.006	5	0	0	0	0	3	8	0	[1]
0.006	7	0	0	0	0	3	8	0	[1]
0.12	14	0	0	0	0	3	8	0	[1]
0.006	1	0	0	20	0	3	8	0	[1]
0.006	3	0	0	20	0	3	8	0	[1]
0.006	5	0	0	20	0	3	8	0	[1]
0.006	7	0	0	20	0	3	8	0	[1]
0.12	14	0	0	20	0	3	8	0	[1]
0.006	1	0	0	30	0	3	8	0	[1]
0.006	3	0	0	30	0	3	8	0	[1]
0.1	5	0	0	30	0	3	8	0	[1]
0.125	7	0	0	30	0	3	8	0	[1]
0.124	14	0	0	30	0	3	8	0	[1]
0.024	1	0	0	50	0	3	8	0	[1]
0.03	3	0	0	50	0	3	8	0	[1]
0.125	5	0	0	50	0	3	8	0	[1]
0.63	7	0	0	50	0	3	8	0	[1]
0.65	14	0	0	50	0	3	8	0	[1]
0.09	0	0	0	50	0	3	8	0	[20]
0.2	0	0	0.5	50	0	3	8	0	[20]
0.16	0	0	1	50	0	3	8	0	[20]
0.16	0	0	1.5	50	0	3	8	0	[20]
0.1	0	0	2	50	0	3	8	0	[20]
0.02	0	0	0	50	0	3	8	0	[26]
0.1	0	0.5	0	50	0	3	8	0	[26]
1	0	5	0	50	0	3	8	0	[26]
1.4	0	0	1.5	50	0	3	8	0	[26]
1	0	0.5	1.5	50	0	3	8	0	[26]
0.8	0	5	1.5	50	0	3	8	0	[26]
1.7	0	0	0	62.5	1	20	2	0	[7]
1.5	0	0	0	65	1	20	2	0	[7]
0.9	0	0	0	67.5	1	20	2	0	[7]
1.3	0	0	0	70	1	20	2	0	[25]
1.3	0	0	0.5	70	1	20	2	0	[25]

1.8	0	0	1	70	1	20	2	0	[25]
1.1	0	0	2	70	1	20	2	0	[25]
2	0	0	0	64	3	20	0	2	[10]
6	1	0	0	64	3	20	0	2	[10]
9	3	0	0	64	3	20	0	2	[10]
11	7	0	0	64	3	20	0	2	[10]
11	14	0	0	64	3	20	0	2	[10]
8	0	0	0	64	3	40	0	2	[10]
10	1	0	0	64	3	40	0	2	[10]
13	3	0	0	64	3	40	0	2	[10]
14	7	0	0	64	3	40	0	2	[10]
15	14	0	0	64	3	40	0	2	[10]
9	0	0	0	64	3	20	2	2	[10]
12	1	0	0	64	3	20	2	2	[10]
14	3	0	0	64	3	20	2	2	[10]
16	7	0	0	64	3	20	2	2	[10]
16.5	14	0	0	64	3	20	2	2	[10]
13	0	0	0	64	3	40	2	2	[10]
15	1	0	0	64	3	40	2	2	[10]
15	3	0	0	64	3	40	2	2	[10]
17	7	0	0	64	3	40	2	2	[10]
18	14	0	0	64	3	40	2	2	[10]
14	0	0	0	64	3	20	4	2	[10]
16	1	0	0	64	3	20	4	2	[10]
17	3	0	0	64	3	20	4	2	[10]
18	7	0	0	64	3	20	4	2	[10]
18.5	14	0	0	64	3	20	4	2	[10]
18	0	0	0	64	3	40	4	2	[10]
18	1	0	0	64	3	40	4	2	[10]
21	3	0	0	64	3	40	4	2	[10]
20.5	7	0	0	64	3	40	4	2	[10]
22	14	0	0	64	3	40	4	2	[10]

Figure S1 shows the compressive strength of predicted and experimental MC loaded samples versus wt% of GO.

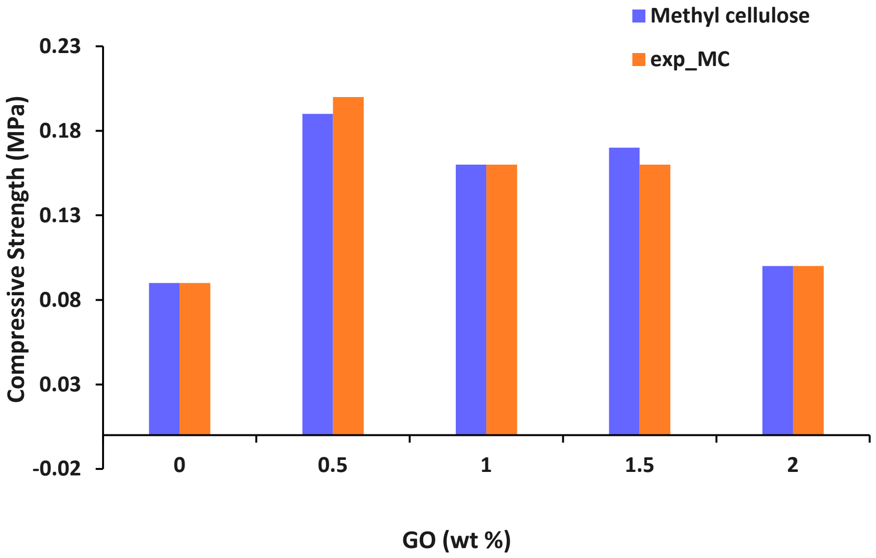

Figure S1. Compressive strength of predicted and experimental MC loaded samples versus wt% of GO (cement phase=50%, % cellulose=4, % chitosan=0).
